# Senescent Changes and Endoplasmic Reticulum Stress May Be Involved in the Pathogenesis of Missed Miscarriage

**DOI:** 10.3389/fcell.2021.656549

**Published:** 2021-06-17

**Authors:** Yunhui Tang, Xinyan Zhang, Yi Zhang, Hua Feng, Jing Gao, Haiyan Liu, Fang Guo, Qi Chen

**Affiliations:** ^1^Department of Family Planning, The Hospital of Obstetrics and Gynecology, Fudan University, Shanghai, China; ^2^The Institution of Obstetrics and Gynaecology, The Hospital of Obstetrics and Gynecology, Fudan University, Shanghai, China; ^3^Department of Obstetrics and Gynaecology, The University of Auckland, Auckland, New Zealand; ^4^Department of Cervical Disease, The Hospital of Obstetrics and Gynecology, Fudan University, Shanghai, China; ^5^Department of Medical Laboratory, The Hospital of Obstetrics and Gynecology, Fudan University, Shanghai, China; ^6^Department of Obstetrics, The Hospital of Obstetrics and Gynecology, Fudan University, Shanghai, China

**Keywords:** placenta, miscarriage, senescence, endoplasmic reticulum stress, extracellular vesicles, misfolded proteins, miRNA sequence

## Abstract

**Background:**

Senescence is involved in many complications of pregnancy. However, whether senescent changes are also associated with missed miscarriage has not been fully investigated.

**Methods:**

The levels of p16, p21, and γH2AX, markers of senescence, were measured in placentas collected from women with missed miscarriage by immunohistochemistry and Western blotting. Levels of misfolded proteins in missed miscarriage placentas or normal first-trimester placenta that had been treated with H_2_O_2_ (100 μM) or extracellular vesicles (EVs) collected from missed miscarriage placental explant culture were measured by fluorescent compound, thioflavin-T. The production of reactive oxygen species (ROS) by missed miscarriage placentas was measured by CellROX^®^ Deep Red.

**Results:**

Increased levels of p16, p21, and γH2AX were presented in missed miscarriage placentas compared to controls. Increased levels of misfolded proteins were shown in missed miscarriage placentas, but not in EVs that were collected from missed miscarriage placentas. The ROS production was significantly increased in missed miscarriage placental explant cultures. Increased levels of misfolded proteins were seen in the normal first-trimester placenta that had been treated with H_2_O_2_ compared to untreated.

**Conclusion:**

Our data demonstrate that there are increases in senescence and endoplasmic reticulum stress and ROS production in missed miscarriage placenta. Oxidative stress and an accumulation of misfolded proteins in missed miscarriage placentas may contribute to the changes of senescence and endoplasmic reticulum stress seen in missed miscarriage placentas.

## Introduction

A missed/silent miscarriage is one type of miscarriage in which the fetus did not form or has died, but the placenta and embryonic tissues still exist. Most of these cases occur in the first trimester of gestation. Although chromosomal abnormalities contribute to the half of missed miscarriage (Vaiman, 2015), other risk factors, including advanced maternal age, previous miscarriage, smoke, obesity, diabetes, and alcohol use, have also been identified (Oliver and Overton, 2014).

A number of studies suggested that defective placentation, including morphological and functional changes in trophoblasts, is associated with many complicated pregnancies, including miscarriage (Hustin et al., 1990; Jauniaux et al., 2003; Jauniaux and Burton, 2005). Recently, a growth of evidence suggested that increased placental oxidative stress represents a common pathogenesis of miscarriage, which may result in impaired trophoblast invasion. These studies indicated that oxidative stress in syncytiotrophoblasts is extensive and is likely a major contributory factor to miscarriage (Hustin et al., 1990; Hempstock et al., 2003; Burton and Jauniaux, 2004). In addition, a recent study reported increased levels of senescence that were triggered by oxidative stress in pathological placentas, such as preeclamptic placentas and post-mature placentas (Cindrova-Davies et al., 2018).

Senescence is the process of gradual deterioration of functional in cells or organs. It is a state of durable growth arrest and irreversible cease proliferation without undergoing cell death. The study has suggested that senescence is triggered by physiological stress and molecular changes when a cell is under stress (Wiley and Campisi, 2016). Cellular senescence can influence the outcomes of a variety of physiological and pathological processes such as cancer and age-related diseases. Cellular senescence can be induced through a number of intrinsic and extrinsic stimuli such as oxidative stress and DNA damage that target a specific population of cells in the tissue. At least three mechanisms, including telomere shortening, upregulation of the CDKN2A locus (which encodes INK4A and ARF), and accumulation of DNA damage, depending on the cell type and the cell culture conditions, are involved in cellular senescence (Collado et al., 2007). Senescence is also relevant to human placental syncytiotrophoblast fusion through activation of cyclin kinase inhibitors such as p21/or p16 (Collado and Serrano, 2010; Cindrova-Davies et al., 2018). In addition, γH2AX, an early cellular response to the induction of DNA double-strand breaks, has been suggested as a sensitive molecular marker of DNA damage (Mah et al., 2010a). Increased levels of γH2AX have been found to promote cell cycle arrest and senescence [reviewed in Mah et al. (2010b)].

Placental dysfunction is associated with the pathogenesis of many complications of pregnancy including miscarriage. Although the underlying mechanism of causing placental dysfunction is still unclear, a number of studies suggested that maternal age and activated natural killer cells contributed to placental dysfunction (Lean et al., 2017; Yougbare et al., 2017). In miscarriage, the development of the placenta and the decidual interface is severely impaired, leading to the early and widespread onset of maternal blood flow and increased oxidative stress. A previous study reported that increased oxidative stress in the placentas is associated with missed miscarriage (Jauniaux and Burton, 2005), and increased oxidative stress induced DNA damage in placentas (Cindrova-Davies et al., 2018). Other studies reported that oxidative stress can disrupt the protein folding process and enhance the production of misfolded proteins (Chong et al., 2017). However, whether the increased oxidative stress induces the damage of DNA in the trophoblast and senescence in miscarriage has not been fully investigated. Missed miscarriage is one of the subtypes of miscarriage. In this study, we investigated whether there are senescent changes in placentas from missed miscarriage.

## Materials and Methods

This study was approved by the ethic committee of The Hospital of Obstetrics and Gynecology of Fudan University, China (reference: 2018-62). All placentas were collected with an informed written patient consent form.

### Tissue Collection and Preparation

Placentas were collected from 25 women with missed miscarriage (9–12 weeks of gestation), who admitted to the family planning clinic in The Hospital of Obstetrics and Gynecology of Fudan University between October 2018 and January 2019. In addition, 25 gestation-matched placentas as controls were collected from healthy pregnancies with elective surgical terminations at random. There was no statistical difference in maternal age, parity, gravida, and history of elective surgical terminations between women with missed miscarriage and controls (*p* > 0.05).

### Reagents

Rabbit anti-human p16 and mouse anti-γH2AX monoclonal antibody (phosphor S139) were purchased from Abcam (Shanghai, China). Rabbit anti-human p21 monoclonal antibody was purchased from Cell Signaling, China. Ready-to-use streptavidin/peroxidase complex reagent (biotinylated anti-mouse/rabbit IgG, Vectastain Elite ABC HRP kit) was purchased from Vector Laboratories, China. CellROX Deep Red Reagent was purchased from Life Technologies (Shanghai, China). Thioflavin-T (ThT) was purchased from Sigma-Aldrich (Shanghai, China).

### Immunohistochemistry

Placentas after surgical uterine evacuation from women with missed miscarriage or from women who had elective surgical terminations were collected (*n* = 25, each). After removal of the decidua, the placental villi were then fixed with 4% paraformaldehyde (PFA) and were embedded in paraffin for further immunohistochemistry analysis. The expressions of p16, p21, and γH2AX (a marker of DNA damage) in placentas were measured by immunohistochemistry on paraffin-embedded sections. In some experiments, healthy first-trimester placentas (*n* = 4) that had been treated with H_2_O_2_ for 18 h were sectioned, and the expression of γH2AX was then measured. Briefly, antigen retrieval was performed by treatment with citric acid (pH 6.0) for 20 min. Non-specific antibody binding was blocked by incubating with 10% normal goat serum for 20 min. Rabbit anti-human p16 (1:50, Abcam) or rabbit anti-human p21 monoclonal antibody (1:50, Cell Signaling, China) or mouse anti-γH2AX monoclonal antibody (phosphor S139) (1:1,000, Abcam) was added for 1 h at room temperature. Sections were then washed with phosphate-buffered saline (PBS) and incubated with ready-to-use streptavidin/peroxidase complexes reagent (biotinylated anti-mouse/rabbit IgG, Vectastain Elite ABC HRP kit; Vector Laboratories) for 10 min. After washing with PBS, sections were then incubated with 3,3-diaminobenzidine for visualization. Sections were then counterstained for 2 min with hematoxylin. Negative controls were performed as above but omitting the primary antibody.

Semiquantitative analysis of the immunohistochemistry result was performed by the strongness of staining, which was scored by two independent authors (QC and YT). Strong staining was scored as 3 points, moderate staining was scored 2 points, and weak staining was scored 1 point compared to the negative control. Data show mean and standard deviation (SD).

### Measurement of Levels of p16, p21, and γH2AX by Western Blotting

The relative protein levels of p16, p21, and γH2AX in placentas from missed miscarriage or controls were also measured by Western blotting. Proteins from missed miscarriage placentas (*n* = 5) or control placentas (*n* = 5) were extracted with RIPA buffer, and all samples (20 μg of total protein) were loaded on 12% sodium dodecyl sulfate–polyacrylamide gel electrophoresis gels, electrophoresed, and then transferred to nitrocellulose membranes. Non-specific binding was blocked by incubating membranes in 3% bovine serum albumin in PBS-T for 1 h at room temperature, and then membranes were incubated with rabbit anti-human p16 (1:50) or mouse anti-γH2AX monoclonal antibody (1:200) or rabbit anti-human p21 monoclonal antibody (1:50) in blocking solution for 2 h at room temperature. After washing with PBS-T three times, the membranes were incubated with goat anti-rabbit or mouse secondary antibody (1:2,000) for 1 h at room temperature. After washing with PBS-T, the membranes were incubated with Amersham^TM^ ECL^TM^ Prime Western blotting detection reagent. Chemiluminescence of the membranes was detected by Image Quant LAS3000. Protein levels of p16, p21, and γH2AX were analyzed relative to the β-actin loading control. Semiquantitative analysis of the Western blots after normalization to levels of β-actin was performed by measuring the density of the band with ImageJ. Data show mean and SD.

### First-Trimester Placental Explant Culture

Healthy first-trimester placental explants (*n* = 5) from elective surgical terminations were cultured in Advanced DMEM/F12 in the presence or absence of H_2_O_2_ (100 μM) for 18 h. The explants were then sectioned for measuring misfolded proteins later.

### Collection of Placental Micro–Extracellular Vesicles and Nano–Extracellular Vesicles

Placental micro– and nano–extracellular vesicles (EVs) were collected from placentas from women with missed miscarriage (*n* = 6) and controls (*n* = 6) as described previously (Abumaree et al., 2006; Chen et al., 2006; Tong et al., 2016). Briefly, placental explants (approximately 400 mg wet weight) were dissected and then were cultured in Netwell^TM^ culture inserts (400-μm mesh), suspended in 12-well culture plates for 18 h at 37°C in Advanced DMEM/F12 containing 2% fetal bovine serum (FBS) in an ambient oxygen atmosphere containing 5% CO_2_. The Netwell^TM^ inserts (containing the explants) were then removed from the culture wells, and conditioned media were aspirated, and then macro-EVs were removed by centrifuging at 2,000*g* for 5 min. The supernatant was then centrifuged at 20,000*g* for 1 h for collection of micro-EVs. The supernatant was further centrifuged at 100,000*g* for 1 h for collection of nano-EVs (Avanti J30I Ultracentrifuge, JA 30.50 fixed angle rotor, Beckman Coulter).

### Measurement of Misfolded Proteins

Misfolded proteins in placentas from women with missed miscarriage (*n* = 6) or controls (*n* = 6) were measured using the fluorescent compound, ThT, as described previously (Beriault and Werstuck, 2013). Briefly, placentas were sectioned and were fixed with 4% PFA for 5 min in room temperature and were then washed with PBS. Sections were then stained with fluorescent compound ThT (500 μM) for 3 min at room temperature. After PBS washing, sections were then counterstained with DAPI for 1 min. The sections were then examined by fluorescent microscope under the same setting (ECLISPE Ni-E motorized fluorescent microscope, Nikon, Japan). In some experiments, the levels of fluorescent compound ThT were also measured in healthy first-trimester placental explants that had been treated with H_2_O_2_ (100 μM) for 18 h.

The levels of fluorescent compound ThT in placental micro- or nano-EVs that were collected from women with missed miscarriage (*n* = 6) or controls (*n* = 6) were also measured. Placental micro- and nano-EVs were incubated with ThT (5 μM) for 10 min at room temperature and then were read in a fluorescent plate reader at 485 nm (Synergie 2, BioTek), following a previously study (Beriault and Werstuck, 2013).

### Reactive Oxygen Species Production

The production of reactive oxygen species (ROS) in placental explants from women with missed miscarriage and controls (*n* = 6, each) was measured using CellROX Deep Red Reagent following the manufacturer guideline. Briefly placental explants (approximately 400 mg wet weight) were dissected and then were cultured in 12-well culture plate in Advanced DMEM/F12 with 2% FBS in an ambient oxygen atmosphere containing 5% CO_2_, with the reagents (1:100). The cellular ROS production was then measured at 30 min and 3 h by a fluorescent plate reader at 665 nm (Synergie 2, BioTek).

### Statistical Analysis

Analysis of ROS production or level of misfolded proteins in placental micro- or nano-EVs between missed miscarriage and controls or semiquantitative analysis of the Western blots or the immunohistochemistry images was assessed by *t* test (non-parametric) using the Prism software package. *p* < 0.05 was considered as statistically significant.

## Results

### Increased Expression of Senescence Markers in Placenta From Missed Miscarriage

**FIGURE 1:**
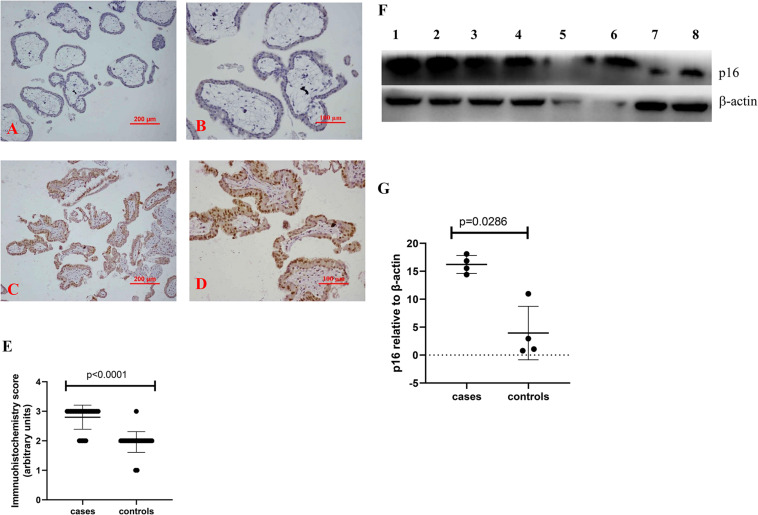
Representative immunohistochemical staining showing that the levels of p16 were increased in missed miscarriage placentas [**(C)** low magnification, **D:** high magnification] compared to healthy first-trimester placentas [**(A)** low magnification, **B** high magnification]. Semiquantitative analysis of the immunohistochemistry images showing a significantly increased expression of p16 in placentas from missed miscarriage **(E)**. Representative Western blot **(F)** demonstrating that the levels of p16 were significantly increased in missed miscarriage placentas (lanes 1–4) compared to controls (lanes 5–8), measured by a semiquantitative analysis **(G)**.

**FIGURE 2:**
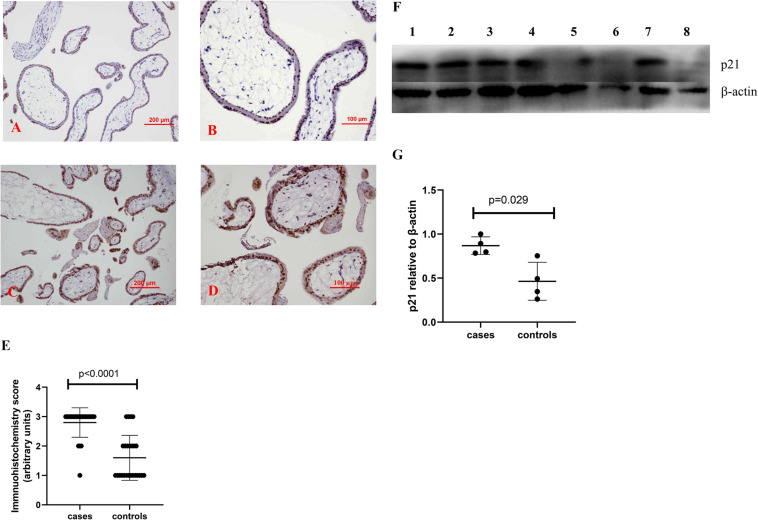
Representative immunohistochemical staining showing that the levels of p21 were increased in missed miscarriage placentas [**(C)** low magnification, **D:** high magnification] compared to healthy first-trimester placentas [**(A)** low magnification, **B:** high magnification]. Semiquantitative analysis of the immunohistochemistry images showing a significantly increased expression of p21 in placentas from missed miscarriage **(E)**. Representative Western blot **(F)** demonstrating that the levels of p21 were significantly increased in missed miscarriage placentas (lanes 1–4) compared to controls (lanes 5–8), measured by a semiquantitative analysis **(G)**.

To investigate whether there is a change in senescent markers in placentas from missed miscarriage, we examined the expression of p16 and p21, which are markers of senescence. Compared to control placentas (Figures 1A,B, 2A,B), a representative image showed that the expression of p16 (Figures 1C,D), or p21 (Figures 2C,D) was predominantly increased in syncytiotrophoblasts in placentas from missed miscarriage. Semiquantitative analysis of the immunohistochemistry images showed the increased expression of p16 or p21 in placentas from missed miscarriage (Figures 1E, 2E, *p* < 0.001) compared to the controls.

**FIGURE 3:**
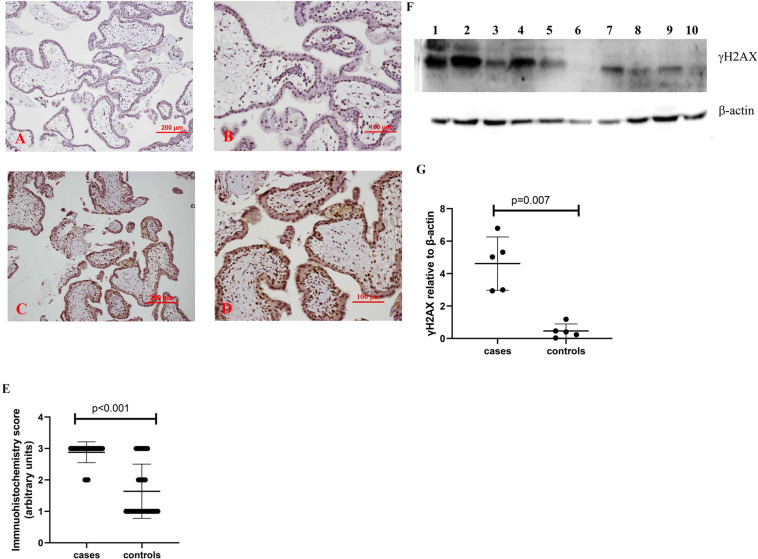
Representative immunohistochemical staining showing that the γH2AX was increased in missed miscarriage placentas [**(C)** low magnification, **D:** high magnification] compared to healthy first-trimester placentas [**(A)** low magnification, **B:** high magnification]. Semiquantitative analysis of the immunohistochemistry images showing a significantly increased expression of γH2AX in placentas from missed miscarriage **(E)**. Representative Western blot **(F)** demonstrating that the levels of γH2AX were significantly increased in missed miscarriage placentas (lanes 1–5) compared to controls (lanes 6–10), measured by a semiquantitative analysis **(G)**.

In addition, compared to control placentas (Figures 3A,B), a representative image showed that the expression of γH2AX was increased in syncytiotrophoblasts in placentas from missed miscarriage (Figures 3C,D). Semiquantitative analysis of the immunohistochemistry images showed the increased expression of γH2AX in placentas from missed miscarriage compared to the controls (Figure 3E, *p* < 0.001).

The significantly increased protein levels of p16, p21, and γH2AX placentas from missed miscarriage were also confirmed by Western blotting (Figures 1F,G, 2F,G, 3F,G).

### Increased Misfolded Proteins in Placentas From Missed Miscarriage

**FIGURE 4:**
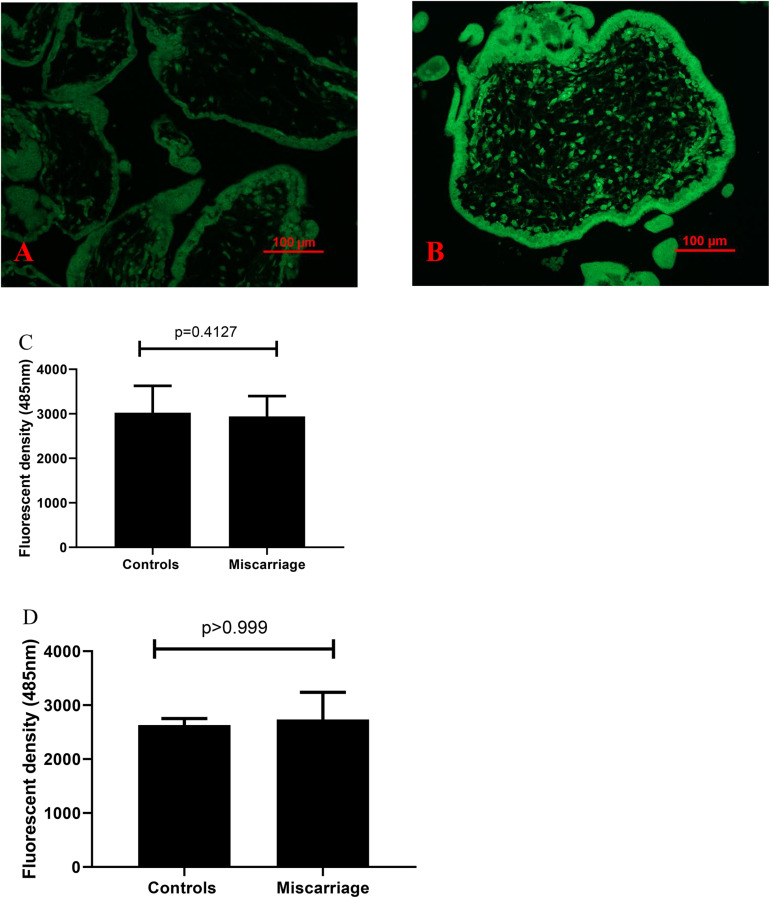
Representative fluorescent compound ThT staining image of a thin section of a placenta showing that the intensity of ThT (green) was increased in placentas from missed miscarriage **(B)** compared to healthy first-trimester placentas **(A)**. ThT fluorescent intensity analysis showing no changes in fluorescent intensity in both placental micro-EV [**(C)**, *p* = 0.4127] and nano-EVs [**(D)**, *p* > 0.999] collected from women with missed miscarriage or controls.

Compared to controls, the fluorescent compound ThT intensity was enhanced in placentas from missed miscarriage (Figures 4A,B). We then measured the intensity of fluorescent compound ThT in placental micro- and nano-EVs that were collected from women with missed miscarriage and controls. There was no difference in the fluorescent compound ThT intensity in both placental micro-EVs (Figure 4C, *p* = 0.4127) and nano-EVs (Figure 4D, *p* > 0.999) between placentas from missed miscarriage and controls.

**FIGURE 5:**
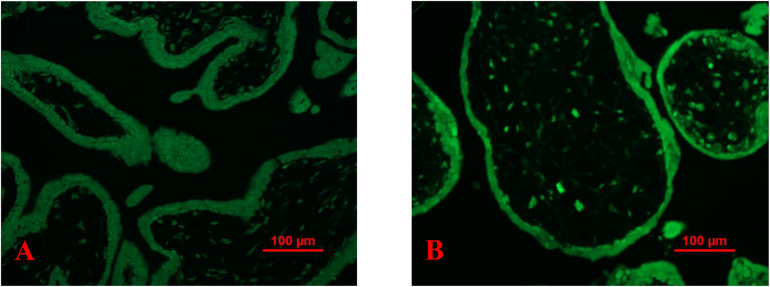
Representative fluorescent compound ThT staining image of a thin section of a placental explant showing that the intensity of ThT (green) was increased in placentas that had been treated H_2_O_2_ **(B)** compared to untreated **(A)**.

We further examined the fluorescent compound ThT intensity in healthy first-trimester placental explants that had been treated with H_2_O_2_. Compared to untreated explants, the levels of the fluorescent compound ThT intensity were increased in healthy first-trimester placental explants that had been treated with H_2_O_2_ treated (Figures 5A,B).

In this study, we also confirmed the increased levels of γH2AX in first-trimester placental explants that had been treated with H_2_O_2_ compared to untreated (data not shown) (Cindrova-Davies et al., 2018).

### Increased ROS Production in Placenta From Missed Miscarriage

**FIGURE 6:**
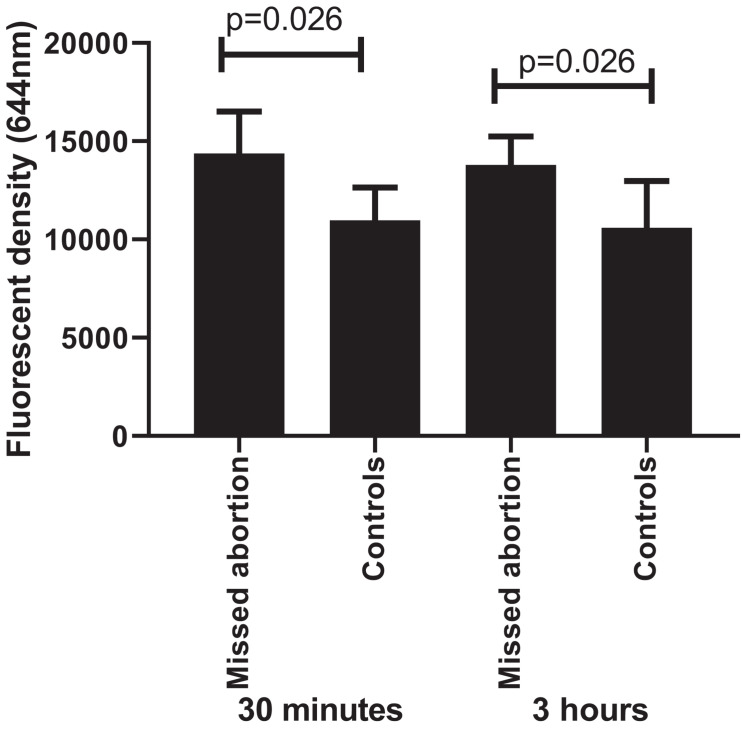
The fluorescent intensity of ROS production showing a significant increase in placental cultures that from missed miscarriage compared to controls, both at 30 min and 3 h (*p* = 0.026, *p* = 0.026, respectively). However, there was no difference in the levels of ROS production between 30 min and 3 h in placental cultures from missed miscarriage (*p* = 0.818).

A previous study reported that oxidative stress refers to elevated intracellular levels of ROS that cause damage to lipids, proteins, and DNA [reviewed in Schieber and Chandel (2014)]. Compared to controls, the ROS production was significantly higher in placental explant culture than that from missed miscarriage after 30 min and 3 h (Figure 6, *p* = 0.026). However, there was no difference in the levels of ROS production among the time points (*p* = 0.818).

## Discussion

The underlying cause in approximately half of miscarriages involves chromosomal abnormalities, suggesting that other mechanisms are also involved in the causes of miscarriage. In our current study, we investigated whether changes of senescence are associated with missed miscarriage. We found that the levels of p16 and p21, as well as γH2AX, were increased in placentas from missed miscarriage, one of the subtypes of miscarriage. In addition, we also found that there were increased misfolded proteins in placentas from missed miscarriage, as well as higher levels of ROS production from placentas from missed miscarriage. Increased oxidative stress may contribute to the changes of senescence and the increased misfolded proteins in missed miscarriage.

Although the molecular signals that are associated with triggering senescence vary, the common molecular markers used to describe senescent cells include telomere shortening, accumulation of DNA damage, increased levels and/or activity of p21, p16, and p19, loss of nuclear HMGB, and enhanced misfolded proteins [reviewed in Cha and Aronoff (2017)]. A number of studies reported that there is an increase in senescent cells in placenta across the gestational age as evidenced by the increased levels of p16 or p21 expression and SA-β-Gal activation (Bonney et al., 2016; Cindrova-Davies et al., 2018). These studies suggested that senescent cells are also present during the early stage of pregnancy that may support embryo development. However, accumulation of senescent cells during pregnancy could cause complications of pregnancy. A recent study reported the increased levels of p16, p21, and γH2AX in post-mature placentas and pathological placentas such as preeclampsia and fetal growth restriction (FGR) (Cindrova-Davies et al., 2018), suggesting senescence is involved in the pathogenesis of complications of pregnancy. Whether changes of senescence are also associated with miscarriage has not been fully investigated. In our current study, we found that both expressions of p16 and p21 were increased in syncytiotrophoblast of placentas from missed miscarriage. In addition, increased levels of γH2AX, a DNA damage marker, were also observed in placentas from missed miscarriage, suggesting that accumulation of DNA damage is present in placentas from missed miscarriage. Our data therefore suggest that changes of senescence could also be associated with the pathogenesis of missed miscarriage.

Cellular senescence is triggered by a number of intrinsic and extrinsic stimuli or stressors. Indeed, pregnancy itself can communicate stress such as oxidative stress and hypoxic stress and can affect placental development. However, these stressors in some stage can impair placental development, resulting in complication of pregnancies such as FGR and preeclampsia. A recent study reported that peroxiredoxin (Prdx) 2, an antioxidant protein, is associated with recurrent miscarriage (Wu et al., 2017). Knockdown of Prdx 2 increased the expression of p21 in trophoblasts that consequently caused miscarriage (Wu et al., 2017). A study indicated that oxidative stress, one of the types of stimuli for inducing senescence, causes cell cycle arrest [reviewed in Pluquet et al. (2015)]. Consistent with a previous study (Cindrova-Davies et al., 2018), in our current study, we also found that oxidative stress induced DNA damage in placental explants. Although placental pathologies are not well addressed in missed miscarriage, a previous study reported that increased oxidative stress in placental tissue is associated with missed miscarriage (Jauniaux and Burton, 2005). Oxidative stress refers to elevated intracellular levels of ROS that cause damage to lipids, proteins, and DNA [reviewed in Schieber and Chandel (2014)]. In our current study, we found that placentas from missed miscarriage produced higher levels of ROS compared to healthy placentas. Our data may suggest that oxidative stress–associated changes of senescence are involved in the pathogenesis of missed miscarriage.

Studies reported that increased oxidative stress linked to endoplasmic reticulum (ER) stress in a number of diseases [reviewed in Chong et al. (2017)]. Oxidative stress induces ER stress, resulting in the accumulation of misfolded proteins in ER and hence becomes toxic and detrimental to cell survive (Pluquet et al., 2015). ER stress can reduce the production of functional proteins and lead to apoptosis [reviewed in Chong et al. (2017)]. Placental ER stress has been found to be raised in a number of pregnancy complications, such as FGR and early onset preeclampsia (Yung et al., 2008, 2014). In addition, placental apoptosis is also reported to be associated with miscarriage (Cinar et al., 2012; Atia, 2017). In our current study, we found increased levels of fluorescent compound ThT, one of the methods for measuring misfolded proteins in placenta from missed miscarriage. Furthermore, when normal first-trimester placental explants were treated with oxidative stressor H_2_O_2_, we also found the increased levels of misfolded proteins in placental explants. A study suggested that misfolded proteins can lead to the generation of oxidative stress in the form of ROS (Gregersen and Bross, 2010). It is well reported that ER stress–induced ROS production medicates a number of diseases. In our current study, we also found higher levels of ROS production from missed miscarriage placentas. However, our data show that the levels of misfolded proteins in placental EVs collected from placental explants from women with missed miscarriage were not different to controls, suggesting those potential dangerous proteins were not exported into EVs, and consequently, there was an accumulation of misfolded proteins in missed miscarriage placentas. Taken together, our data suggest that increased ER stress in the placentas but not released through EVs may be involved in the pathogenesis of missed miscarriage.

A growth of evidence suggested that ER stress is associated with the process of senescence in a number of diseases (Pluquet et al., 2015; Liu et al., 2017; Yuan et al., 2017), because senescence is a stress response. However, it is still unclear whether senescence is causative of ER stress or a consequence of ER stress. It is also unclear whether the severity of ER stress has a different role on the consequence of senescence [reviewed in Pluquet et al. (2015)]. However, ER stress can concomitantly occur with senescence and be involved in the onset or maintenance of senescent features [reviewed in Pluquet et al. (2015)].

In conclusion, in our current study, we found the changes of senescence, increased levels of misfolded proteins, and increased ROS production in placentas from missed miscarriage. Oxidative stress contributed to the changes of senescence. Our data suggest that senescence may be involved in the pathogenesis of missed miscarriage. Our study may provide some novel insights of the underlying mechanism of missed miscarriage, in addition to chromosomal abnormalities.

## Data Availability Statement

The raw data supporting the conclusions of this article will be made available by the authors, without undue reservation.

## Ethics Statement

The studies involving human participants were reviewed and approved by the Ethic Committee of The Hospital of Obstetrics and Gynecology of Fudan University, China (reference: 2018-62). The patients/participants provided their written informed consent to participate in this study.

## Author Contributions

YT: performed the experiments. XZ and JG: assisted to perform the experiments. YZ, HF, and HL: assisted to data analysis. FG and QC: design the study and wrote up the manuscript. All authors were involved in the manuscript preparation.

## Conflict of Interest

The authors declare that the research was conducted in the absence of any commercial or financial relationships that could be construed as a potential conflict of interest.
